# Expression and clinical significance of cyclin kinase subunit 2 in colorectal cancer

**DOI:** 10.3892/ol.2013.1456

**Published:** 2013-07-11

**Authors:** MINHAO YU, MING ZHONG, ZHONGDONG QIAO

**Affiliations:** Department of Surgery, Renji Hospital, Shanghai Jiaotong University, Shanghai 200127, P.R. China

**Keywords:** cyclin kinase subunit 2, colorectal cancer biomarker

## Abstract

The overexpression of cyclin kinase subunit 2 (CKS2) is tightly correlated with tumor aggressiveness and prognosis in various malignancies, including gastric, breast, liver and prostate cancer. However, whether CKS2 is upregulated in colorectal cancer (CRC) remains unknown. The aim of the present study was to analyze CKS2 expression levels in CRC, and to determine the clinical diagnostic and prognostic values of CKS2 overexpression in CRC patients. CKS2 expression was analyzed at the mRNA and protein levels by quantitative (q)PCR and western blot analysis. CKS2 expression was significantly upregulated in CRC compared with the adjacent non-cancer and normal colorectal tissues. The overexpression of CKS2 was correlated with poor differentiation and the pathological stage. In addition, CKS2 overexpression was correlated with aggressive tumor progression in CRC, which indicated that CKS2 may serve as a good CRC biomarker.

## Introduction

Colorectal cancer (CRC), one of the most common primary malignancies, demonstrates molecular heterogeneity during its development and progression (1). The prognosis of patients with CRC is commonly determined by traditional clinicopathological factors, including tumor grade and lymph node status (2,3). Nevertheless, patients may have significantly different clinical outcomes despite the exhibition of similar clinicopathological features (4). Although serum carcinoembryonic antigen (CEA) has long been regarded as the most significant and common biomarker for CRC, there are limitations to the sole use of the CEA level for the early diagnosis and prognosis of CRC (5). Therefore, the identification of novel gene expression that is altered in CRC may aid the understanding of the mechanisms of tumorigenesis, the development of diagnostic biomarkers, the prediction of the clinical prognosis and the design of targeted therapies.

The cyclin kinase subunit (CKS) proteins, which consist of CKS1 and CKS2 in vertebrates, are highly conserved molecules in eukaryotes. These proteins share 81% amino acid sequence homology (6). The overexpression of CKS1 has been demonstrated to be correlated with poor survival rates in patients with breast, colorectal, prostate and renal cancer (7–10). There is accumulating evidence that CKS2 expression, similar to that of CKS1, is upregulated in a variety of malignant tumors, including those of the prostate, bladder and liver (10–12). However, whether CKS2 is overexpressed in CRC remains unclear.

The present study aimed to show that CKS2 expression was significantly upregulated in CRC, and that it was correlated with certain clinical features of CRC. The results suggested that the expression level of CKS2 may have a diagnostic and prognostic value for patients with CRC.

## Materials and methods

### Patients and specimens

As approved by the ethics committee of Renji Hospital (Shanghai Jiaotong University School of Medicine, Shanghai, China), colorectal cancer samples were obtained from 30 patients who underwent routine surgery for CRC at the Department of Surgery between 2010 and 2012. Patients were recruited immediately following surgery and samples of CRC, adjacent non-cancer and normal colorectal tissues were collected at that time. None of the patients had received any pre-operative treatment, including radiation or chemotherapy. Clinical data were recorded and the pathological classification was performed according to a staging system previously described (2). The tissues were immediately placed in TRIzol reagent for the extraction of RNA and protein. Written informed consent was obtained from all patients.

### RNA extraction and quantitative (q)PCR analyses

Tissues were lysed and the total RNA was isolated using TRIzol reagent (Invitrogen Life Technologies, Carlsbad, CA, USA), according to the manufacturer’s instructions. Following quantification of the RNA, a sample containing 2 μg RNA was annealed to the oligo(dT) at 65°C for 5 min and cooled at −4°C for 2 min. A total volume of 20 ml was used for the reverse transcription (RT) reaction; this contained RT-buffer, RNasin, reverse transcriptase, dNTPs and RNA-oligo(dT) mixtures. The PCR reaction was conducted at 42°C for 60 min, and the following primers were used: CKS2 forward, 5′-GCTCTTCGCGCTCTCGTTTCATTT-3′ and reverse, 5′-ACTCTGTTGGACACCAAGTCTCCT-3′. The PCR reactions were terminated subsequent to 35 cycles. For the PCR quantitation, the SYBR, primers and cDNA were mixed, and the reaction was performed for 40 cycles using the MJ Research PTC-l00 Thermal Cycler system (Bio-Rad, Hercules, CA, USA). The data were normalized with the glyceraldehyde3-phosphate dehydrogenase (GAPDH) housekeeping gene. All primers were custom-synthesized by Sangon Biotech (Shanghai) Co., Ltd. (Shanghai, China).

### Western blot analysis

The total protein was extracted from ~0.5 g frozen tissue using radioimmunoprecipitation assay (RIPA) buffer (Beyotime, Shanghai, China). Aliquots containing 30 mg protein were subjected to sodium dodecyl sulfate-polyacrylamide gel electrophoresis and electroblotted onto a polyvinylidene difluoride membrane (Amersham Biosciences AB, Uppsala, Sweden) for western blot analyses for 2 h. Following incubation with 5% skimmed milk for 2 h, the membranes were incubated with the primary antibody [anti-CKS2, dilution of 1:3,000 in Tris-buffered saline and 0.1% Tween 20 (TBST)] for 1 h at room temperature. Each membrane was then washed three times with TBST for 10 min followed by incubation with the secondary antibody goat anti-mouse IgG-HRP (Santa Cruz Biotechnology Inc., Santa Cruz, CA, USA) (1:10,000–30,000 dilution) for 1 h. Following three 10-min washes with TBST, the specifically bound antibodies were detected with the Enhanced Chemiluminescence (ECL) kit (MultiScience Biotech Co., Shanghai, China), according to the manufacturer’s instructions. The intensity of the bands was quantified usign the Tanon GIS system (Tanon, Shanghai, China) and the data were normalized to the GAPDH loading controls.

### Statistical analysis

All data were processed with SPSS 13.0 software (SPSS, Inc., Chicago, IL, USA). The Kruskal-Wallis non-parametric test was used to analyze the correlations between CKS2 mRNA expression and various clinicopathological features. P<0.05 was considered to indicate a statistically significant difference, and was calculated by the two-tailed test.

## Results

### Correlations between CSK2 expression and clinicopathological features

The clinical findings are summarized in Table I. A total of 14 males (46.7%) and 16 females (53.3%), with ages ranging between 27 and 81 years (median, 62 years; mean, 58.7 years) were recruited into the study. Thirteen patients presented with rectal cancer and 17 with colon cancer. The post-operative pathological classifications were performed according to the NCCN Guidelines Version 2.2012, and included 15 patients (50.0%) each in stages I and II.

### Expression of CKS2 is elevated at the mRNA and protein levels in CRC

**Figure 1:**
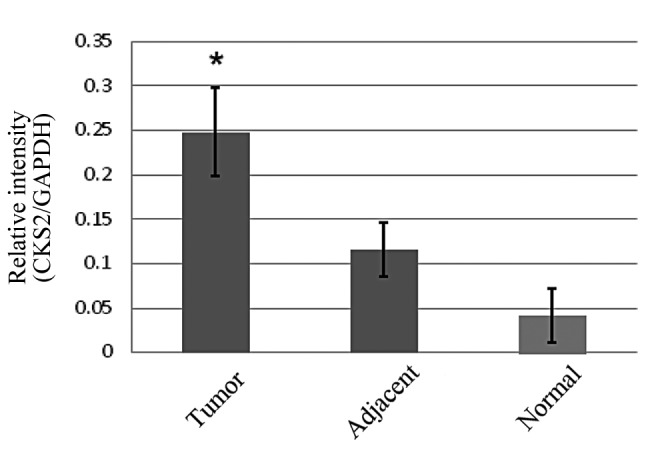
Overexpression of cyclin kinase subunit 2 (CKS2) at the mRNA level in different types of tissue. Total RNA samples were extracted from colorectal cancer (CRC), adjacent non-cancer and normal colorectal tissues. Expression of CKS2 was assessed by quantitative (q)PCR. Data were normalized to glyceraldehyde-3-phosphate dehydrogenase (GAPDH). ^*^P<0.05 vs. control.

**Figure 2:**
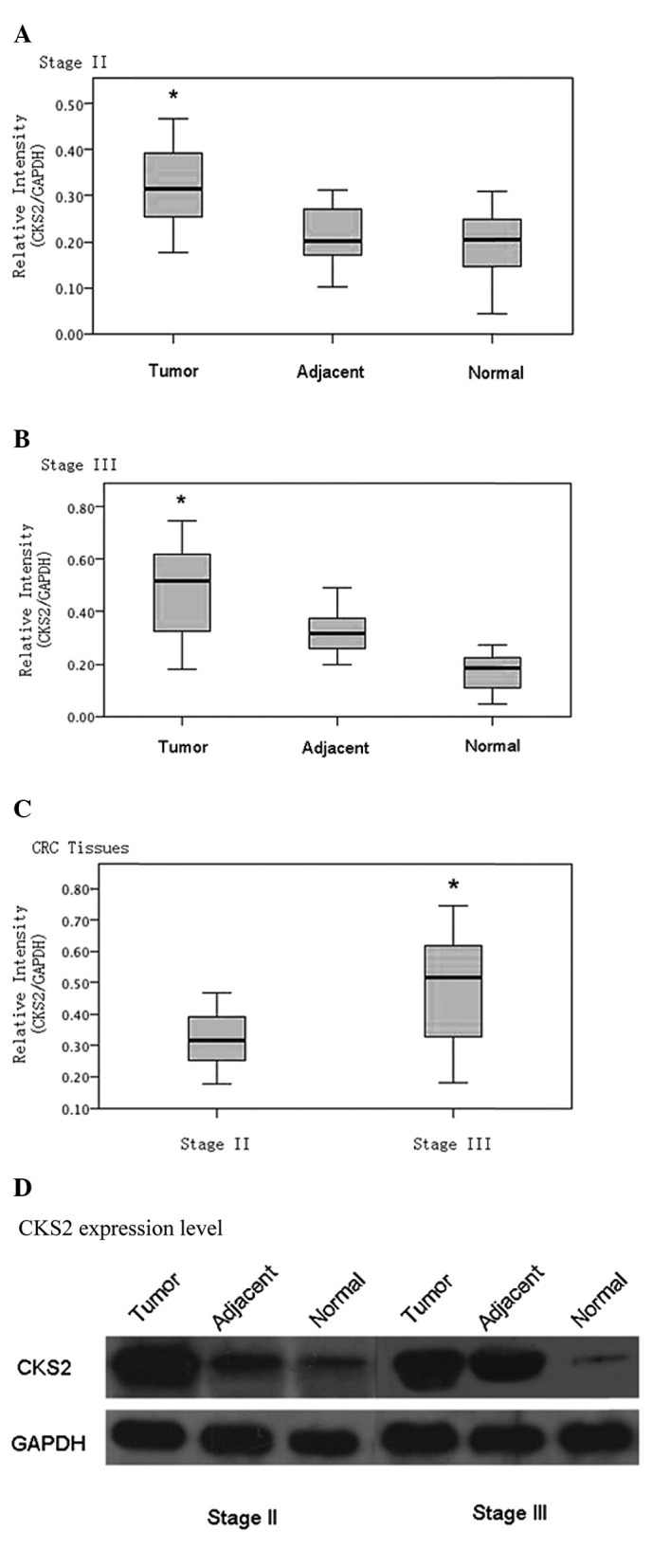
Western blot analyses were used to determine cyclin kinase subunit 2 (CKS2) protein expression levels in tumor tissues from stage II and III colorectal cancer patients and adjacent non-cancer and normal colorectal tissues. (A) In stage II CRC, CKS2 protein levels were significantly increased compared with the normal and adjacent tissues. ^*^P<0.05 vs. control. (B) In stage III CRC, CKS2 protein levels were significantly increased in the tumor and adjacent tissues compared with the normal tissue. ^*^P<0.05 vs. control. (C) Tumor tissues from stage III CRC patients demonstrated significantly higher CKS2 protein levels than those from stage II patients. ^*^P<0.05 vs. control. (D) The proteins extracted from the tissues were subjected to western blot analyses as indicated. Glyceraldehyde-3-phosphate dehydrogenase (GAPDH) was used as the loading control.

To determine whether CKS2 was overexpressed in CRC, the mRNA and protein levels of CKS2 were measured in the tumor and adjacent non-tumor tissues, as well as in the normal colorectal tissue. qPCR analyses revealed that the mRNA levels of CKS2 were significantly increased in the CRC tissue compared with the adjacent non-tumor and normal colorectal tissues (Fig. 1). Western blot analyses demonstrated that the expression of the CKS2 protein was also upregulated in the CRC tissue samples, as shown in Fig. 2.

### Overexpression of CKS2 is correlated with the aggressive behavior of CRC

To further examine the clinicopathological relevance of CKS2 overexpression in CRC, CKS2 expression was analyzed in correlation with pathological features of tumors. The results revealed that the overexpression of CKS2 at the protein level was significantly correlated with tumor size, differentiation and pathological tumor node metastasis (pTNM) stage (Table I). No significant correlation was detected between CKS2 overexpression and other clinicopathological features, such as patient age and gender or tumor location.

## Discussion

The CKS proteins, including CKS1 and CKS2, are essential components of cyclin/cyclin-dependent kinase (CDK) complexes that are involved in the regulation of cell cycle progression. The CKS proteins exhibit 81% amino acid sequence identity (13). The dysregulation of the CKS proteins and other cell cycle-related regulators, including the cyclins and CDKs, has been demonstrated to be associated with several types of tumors (14,15).

The present study identified that CKS2 was overexpressed at the mRNA and protein levels in CRC tissues in comparison with the adjacent non-cancer and normal colon tissues. However, the opposite was observed in certain samples. This may occur in clinical practice due to the lack of a clear definition of the adjacent non-tumor tissue. Furthermore, these data clearly demonstrated that the overexpression of CKS2 was significantly correlated with tumor differentiation and lymph node metastasis, which may have contributed to the development of CRC. However, as the complete course of the CRC patients was not available, a Kaplan-Meier survival analysis could not be conducted.

Although the level of CKS2 expression was significantly higher in the tumor tissue than in the adjacent non-cancer and normal colorectal tissues, there were certain discrepancies in the correlation between CKS overexpression at the mRNA and protein levels and lymph node metastasis. These discrepancies may be attributed to the relatively small sample size in the present study. Different translation efficiencies or stabilities of the protein in the tumor tissues may also have caused the discrepancies in the results. In addition, genetic and epigenetic factors, including DNA methylation, genetic mutation and abnormal post-transcriptional regulation, may have contributed to the variation in the results (16). Further studies are required to clarify this issue.

In conclusion, to the best of our knowledge, this is the first study to demonstrate that CKS2 is overexpressed in CRC. The results suggested that the aberrant expression of CKS2 may contribute to the development and progression of CRC, and that CKS2 expression patterns may be of diagnostic and prognostic value for CRC patients.

## Figures and Tables

**Table I:** Correlations between CKS2 expression and clinicopathological features in CRC.

Characteristics	No. of patients	CKS2 protein expression
Age (years)		
<50	8	0.152±0.013
≥50	22	0.201±0.024
Gender		
Female	16	0.178±0.019
Male	14	0.192±0.032
Tumor diameter (cm)		
<4	12	0.138±0.026
≥4	18	0.214±0.010^a^
Differentiation		
Well	4	0.165±0.021
Moderate	19	0.169±0.019
Poor	7	0.237±0.027^a^
Location		
Rectum	13	0.182±0.016
Colon	17	0.202±0.014
pTNM stage		
II	15	0.116±0.051
III	15	0.248±0.030^a^

a P<0.05.

CKS2 protein expression is presented as the mean ± standard deviation. CKS2, cyclin kinase subunit 2; CRC, colorectal cancer; pTNM, pathological tumor node metastasis.
